# How older adults manage misinformation and information overload - A qualitative study

**DOI:** 10.1186/s12889-024-18335-x

**Published:** 2024-03-21

**Authors:** M. Vivion, V. Reid, E. Dubé, A. Coutant, A. Benoit, A. Tourigny

**Affiliations:** 1 Department of Social and Preventive Medecine, Université Laval, Quebec, Canada; 2 CHU de Québec-Université Laval Research Center, Quebec, Canada; 3 Laboratoire sur la communication et le numérique (LabCMO), Montreal, Canada; 4 Department of Anthropology, Université Laval, Quebec, Canada; 5 Université du Québec à Montréal (UQAM), Montreal, Canada; 6 GDR AREES (Groupe de recherche: Arctique: Enjeux pour l’environnement et les sociétés) du CRNS, Paris, France; 7 Institut sur le vieillissement et la participation sociale des aînés de l’Université Laval, Quebec, Canada; 8 VITAM Centre de recherche en santé durable, Quebec, Canada

**Keywords:** Misinformation, Information overload, social media, informational practices, COVID-19, Older adults, Quebec

## Abstract

**Background:**

The COVID-19 pandemic was characterized by an abundance of information, some of it reliable and some of it misinformation. Evidence-based data on the impact of misinformation on attitudes and behaviours remains limited. Studies indicate that older adults are more likely to embrace and disseminate misinformation than other population groups, making them vulnerable to misinformation. The purpose of this article is to explore the effects of misinformation and information overload on older adults, and to present the management strategies put in place to deal with such effects, in the context of COVID-19.

**Methods:**

A qualitative exploratory approach was adopted to conduct this research. A total of 36 semi-structured interviews were conducted with older adults living in Quebec, Canada. The interviews were fully transcribed and subjected to a thematic content analysis.

**Results:**

Participants said they could easily spot misinformation online. Despite this, misinformation and its treatment by the media could generate fear, stress and anxiety. Moreover, the polarization induced by misinformation resulted in tensions and even friendship breakdowns. Participants also denounced the information overload produced largely by the media. To this end, the participants set up information routines targeting the sources of information and the times at which they consulted the information.

**Conclusions:**

This article questions the concept of vulnerability to misinformation by highlighting older adults’ agency in managing misinformation and information overload. Furthermore, this study invites us to rethink communication strategies by distinguishing between information overload and misinformation.

**Supplementary Information:**

The online version contains supplementary material available at 10.1186/s12889-024-18335-x.

## Background

The COVID-19 pandemic was accompanied by a steady stream of information that was described by the World Health Organization as an “infodemic,” referring to an epidemic of information, some of which was reliable while other items of information were erroneous or false [1]. This information overload refers to a state where and individual’s efficiency in selecting, using, processing, and making sense of information is hampered by the quantity of pertinent and possibly valuable information [2]. Information overload is closely linked to information redundancy, as they mutually increase each other [3]. Information redundancy designates repeated messages within a sequence of received messages [3]. In addition to pertinent information, information overload is further exacerbated by disinformation and misinformation [1]. This state can be accompanied by a feeling of loss of control and anxiety [2]. Information overload has essentially been studied in relation to the issues of misinformation and disinformation, rather than as a separate phenomenon [4, 5]. Disinformation and misinformation refer to incorrect information [6, 7]. While disinformation implies malicious intent, and is associated with fake news and conspiracy theories, for example, misinformation does not imply malicious intent [6, 8]. The distinction therefore lies in the intention to deceive or not, which is difficult to prove. For this reason, the term “misinformation” will be used in this article to refer to information created and disseminated irrespective of any intention to deceive.

Health misinformation refers, more specifically, to “information that is contrary to the epistemic consensus of the scientific community regarding a phenomenon” [9]. Misinformation is most often characterized by a negative tone, the preponderance of anecdotes and personal experiences, the promotion of anti-scientific narratives, and rapid dissemination [9]. In health, misinformation is particularly problematic, as it can lead to misperceptions leading to potentially harmful action [9, 10]. For example, a false connection between the measles-mumps and rubella vaccine and autism led to decrease in vaccination coverage at the end of the 1990s and is still a common reason for vaccine refusal today [11]. In fact, during the pandemic, misinformation first took the the form of calls suggesting the necessity to stock up on certain supplies (e.g, toilet paper, food), and then focused mainly on unproven “treatments” or techniques to prevent infection (drinking water with lemon or coconut oil, probiotics) and then to forms of denials of the data about the number of cases and deaths– or even the existence of the disease [12, 13]. It would also discredit the scientific community and public health authorities, or make it more difficult for people to determine which information to believe, and fuel a sense of panic [14]. More generally, misinformation can lead to various forms of violence (xenophobia, bullying, verbal or physical violence), discrimination such as anti-Asian attitudes and behaviors during the COVID-19 pandemic, and psychological distress [11, 15]. Also, during the pandemic, an increase in uncertainty and a heightened need for new information, especially in healthcare, to address it led to an information overload [14].

Despite this, the effects of misinformation remain unclear, and empirical evidence of its impact on behaviour and attitudes is limited [10]. A systematic review reveals that articles reporting concrete cases of damage caused by misinformation are rather rare [16]. Concerns about misinformation persist, rooted in the belief that it can significantly influence people’s thoughts and behaviors [10]. This, in turn, poses a potential threat to both public health and the integrity of democracy [10].

As social media can rapidly and widely disseminate information, they are frequently mentioned in articles discussing misinformation [13, 17]. In this regard, studies on the dissemination of misinformation indicate that older adults are more “vulnerable” to misinformation, as they tend to subscribe to erroneous messages spread online and are more responsible for their dissemination than other population groups [14, 15, 18–21]. Studies explain this “vulnerability” by the lack of skills needed to find and evaluate online information [15, 22–24]. Some authors associate this lack of skill with a “cognitive deficit” which refers to the decline in cognitive abilities that occurs with aging, [19, 20, 25–28] affecting the ability to distinguish true from false information. This one-size-fits-all approach, which most often groups together adults aged 65 and over, is criticized for neglecting the diversity of uses and experiences of the Internet, and also for denying older adults’ agency, thus perpetuating ageism [29, 30]. Moreover, most studies focused on older adults’ engagement with misinformation come from the realm of politics or the media [19, 31], rather than health-related issues [9, 32]. This raises the question of the transferability of the results from these studies on the vulnerability of older people to health misinformation [9].

The purpose of this article is to explore the effects of misinformation and information overload for older adults, and to present the management strategies put in place to deal with them. This article is part of a larger research project to study the informational practices of Quebec older adults in the context of the COVID-19 pandemic. The general results of this research were presented in another article [33].

## Method

### Research ethics

The study protocol was approved by the Centre intégré universitaire de santé et de services sociaux du Centre-Sud-de-l’Île-de-Montréal (CIUSSS) Ethics Committee (Project 2022 − 829).

### Design and settings

This research employed an exploratory approach based on the principles of the sociology of uses [34]. The relevance of the sociology of uses approach for analyzing informational practices has been established by several studies [35–39]. This approach recontextualizes the uses of information and communication technologies within their social and everyday contexts [40]. The concept of ‘’usages’’ as mobilized by the sociology of uses emphasizes the autonomy of users and their appropriation of technical devices [38]. This approach thus opposes a deterministic view of technology. In this study, participants were questioned about the devices used and their routines associated with information practices (time of day, order in which sources are consulted, activities performed simultaneously). We also made sure to account for the complexity of informational practices, which is essential to consider in accordance with sociology of uses [38]. For instance, the interview framework was designed to inquire about participants’ multiple sources of information and material supports they used [38]. This approach also guided our analysis by focusing on the potentialities related to the agency of individuals when they consume media content. The research project was developed in collaboration with the Conference of the Regional Roundtables for Older Adults in Quebec (CTRCAQ).

### Participants

Participants were recruited through a list created as part of weekly surveys designed to assess adherence to COVID-19-related health measures among the Quebec population. These surveys were conducted by the Institut National de Santé Publique du Québec (INSPQ). More information on the survey methods and limitation is available here [41].The database contained participants’ age, self-identified gender, place of residence, and email addresses, but was not linked with complete answers to the survey. During the surveys, participants could indicate whether they wished to be contacted by other research teams to participate in studies. The research team sent an invitation email to the participants based on criteria such as age, gender, and residency. During the surveys, participants could indicate whether they wished to be contacted by other research teams to participate in studies. The research team sent an invitation email to the participants [41]. The participants had to be aged 60 or older, residing in the province of Quebec, and fluent in either French or English. The participants were informed that the study would delve into how they obtain information during the pandemic, including the sources they use, and how they assess its credibility. Participants received financial compensation of $30. Informed consent, either written or oral, was obtained before each interview.

### Study tool

To comply with preventive measures, the interviews were conducted online during the summer of 2021, 15 months after the start of the pandemic. Lasting approximately one hour, they were conducted in French. Topics covered included: sources of information used, their information needs arising from the pandemic, ways of appropriating information and assessing its validity or credibility, and the impact of this information on their health-related behaviours. Participants were also asked about the change in their information practices as a result of the COVID-19 pandemic, and about how they perceived misinformation. The English interview guide is provided in a supplementary file.

### Data analysis

Interviews were transcribed in full. We conducted a thematic analysis that involves categorizing a given dataset into predetermined themes that accurately represent the analyzed content, tailored to the research objectives [42]. Although the coding was guided by theoretical framework of informational practice, new themes could be added over the course of coding, as in the case of the information overload management strategies theme. Verbatim could be associated with more than one theme. The categorization of verbatim excerpts into different categories was carried out using NVivo software. For this paper, we translated verbatim excerpts from French to English, ensuring the quality of our work by validating it with a certified translator. An initial coding was carried out by A.B, then revised by M.V. A comparison of the two researchers’ coding led to a consensus on the meaning of certain ambiguous extracts and their association with a theme.

## Results

First, we will present the participant profiles. Subsequently, we will highlight the study’s results, organized around four main themes: information sources; effects of misinformation; consequences of information overload; and strategies for managing misinformation and information overload.

### Participant profiles

A total of 36 semi-structured interviews were conducted with Quebec men (*n* = 18) and women (*n* = 18), living in a house or apartment in an urban (*n* = 20) or rural (*n* = 16) setting. Only one participant lived in a seniors’ residence. The majority of participants were aged between 60 and 69 (*n* = 28), with the remainder aged 70 and over. The distribution of participants according to high school, college or university level of education was the same (*n* = 12). Participant demographics are presented in Table 1.

**Table 1:** Participant demographics

	Sociodemographics	Number of participants (n)
Gender		
	Men	18
	Women	18
Age		
	60–69 years old	28
	70 years old and over	8
Geographical region		
	Central regions of Quebec	20
	Other regions of Quebec	16
Education attainment		
	Secondary school diploma	12
	College diploma	12
	University degree	12

### Information sources

During the pandemic, older adults in our study consulted multiple sources of information. Traditional media outlets, including television, were the preferred sources. Social media, especially Facebook, were used more to consult Market Place, for entertainment purposes and to communicate with their loved ones. As one participant had put it:

“With a cousin who lives in the greater Vancouver area, we don’t see her regularly, so we chat about stuff on Facebook. Then, I was faced with some mortality, people who passed away recently, so Messenger or Facebook would allow us to communicate. So it’s more about keeping in touch with people you don’t see very often, or with people you haven’t seen in a long time.” (P 27; 62 years old).

Some sources are more closely associated with the presence of misinformation. For one participant, using social media to stay in touch sometimes meant being exposed to what they perceived as misinformation: “I look at my son, my youngest, who sends me videos, stuff on Facebook… I don’t know where he gets this stuff… no. [sigh] It’s arrogance, I call it fake news.” (P 30; 64 years old).

### The effects of misinformation

According to a number of participants, misinformation was generally easy to detect, as the information often “makes no sense at all,” so that “it’s often almost easy to see” (P 14;71 years old). While most report the harmful effects of this phenomenon (confusion, fear, worry, anxiety), one participant pointed out that misinformation at least had the advantage of potentially raising questions: ‘’Fake news, their only positive aspect is that it can make us question things a bit’’ (P 36; 71 years old).

Although misinformation affected participants, many indicated that it was more detrimental to their loved ones. Firstly, emotional responses such as confusion or fear were mentioned:

“Well, I think it’s a shame because it confuses people. It scares people; you know, I find it, uh, stupid that people invent things like… a friend of mine, she told me “you know, those who get vaccinated will all be dead in two years,” and I said “well yes, well yes.” But in my head I’m thinking… Come on, wake up. That makes no sense, there won’t be anyone left on Earth.” (P 33; 65 years old).

Another participant explained: “When we have our seniors’ meetings. A lot of things are said on social media and they hear it and it scares them. Often, it’s out of fear…”. (P 31; 72 years old).

Exposure to misinformation also led to worry: “I was reading [on social media] at first, then when I saw that it was starting to get me worried, I stopped that too, because it didn’t make sense, on social media, there’s really nothing serious about it.“(P 33;65 years old).

For many, misinformation was generating anxiety, and for some, even aggression, to which exposure to repeated negative information in the media was also contributing:

“We don’t have any hopeful news, it’s always about getting bad news fed into our heads, which creates a climate of anxiety, a climate that I consider unhealthy. There’s a huge level of aggression in Quebec right now, which is totally unacceptable. I blame the news a lot for that, because it creates chaos and enormous stress in society by repeating… non-stop, nothing but bad news. Also, there’s a lot of misinformation circulating and that creates anxiety, anguish and aggression in many communities.” (P 29; 64 years old).

The second type of effect identified and denounced by the participants was the resulting polarization. Misinformation was associated with heated debates between friends. A participant claimed to have occasionally listened to conspiracy theorists but did not like them. They mentioned engaging in debates at times with a politically far-right friend who is associated by the participant with such theories: “I have friends on the right, on the far right, I don’t have many, but I have one, but we often bump into each other, but it’s a good omen, as they say.” (P 9; 65 years old).

On this subject, one participant denounced the term “covidiot” used by the media during the pandemic, which she felt contributed to polarization. This portmanteau, combining the words “covid” and “idiot”, refers to someone who behaves in a stupid way that risks spreading the infectious disease covid-19. The expression is often attributed to individuals who refuse to accept vaccines or question preventive measures. Polarization had, for some, led to broken friendships:

“Apart from that, I’d say we had one friend who we cast aside because she became completely… and I mean completely conspiratorial. It was ridiculous. She was so exaggerated that we didn’t even question it anymore. We respected her, we didn’t laugh in front of her, but the minute she was gone… In fact, in my opinion, it’s mental illness. I don’t know why it’s called mental illness due to fear of the pandemic, but for me it’s clearly that. I’m not a doctor or a psychologist, but… […] She was affected in the head.” (P 18; 64 years old).

### Consequences of information overload

In addition to misinformation, most participants deplored information overload and redundancy in the media. Faced with the repetition of information, participants were “fed up with hearing about it” (P 15; 66 years old) and were irritated. “At first, it was awful. It was just that” (P 22; 63 years old). Another participant lamented the way the media operates under the logic of streaming:

“Also, it’s repetitive […]. In these times, the news is non-stop, so it becomes a show. Then, at a certain point, it’s clear that we’re fed up. It’s become so repetitive […] We’re buried in information. We receive an enormous amount of information.” (P 29; 64 years old).

While information overload was a source of stress for many participants, it was especially the type of information conveyed by the media that seemed responsible. The daily presentation of the number of cases and deaths, as well as the description of situations in hospitals and Long-Term Care Facilities (CHSLDs), were considered particularly anxiety-provoking by participants. As one participant had put it: “When they started mentioning the number of cases per day… When we saw that, well, when we saw that going up, it was a bit stressful as well.” (P 4; 64 years old).

The major concern about the situation in CHSLDs and the treatment of the older adults was also explained by the particular point of view of our participants, some of whom dread the day when they will have to move to this type of facility for people who are not self-sufficient:

“[…] for me personally, it was very stressful to - it seems to me that I was so glued to the TV. Just to find out what was happening, well, as much in the CH [SLD] for the elderly. It certainly made me wonder, in the sense that I’m coming from there. You know, I’m going that way. […]. But it’s worrying. When you look at how they’ve been treated and everything. My God, to think I’m going in that direction. It’s like, it’s not very reassuring.” (P 2; 64 years old).

### Strategies for managing misinformation and information overload

Analysis of the data revealed that participants implement several strategies to limit the effects of misinformation and information overload, or anxiety-provoking information.

First, most of them claimed to limit themselves to so-called reliable sources, meaning for them traditional media such as Radio-Canada and TVA Nouvelles, which for many participants provide access to “real” information and “real” news:

“How can I put this… the real news…, the verified news, comes from television media: TVA or Radio-Canada or RDI. If it’s fake news that comes through them, they’re the ones with a hell of a problem, because they haven’t checked the news. So, in principle, if it comes from Wikipedia, the news […] from another site that isn’t an official site, well, I don’t even pay attention to it. When I get news that doesn’t come from a place that’s known and approved by me, let’s put it this way, it’s quickly passed over, forget it, I don’t even pay attention to it.” (P 32; 64 years old).

To avoid misinformation, most participants did not seek information on social media, which they consider not to be reliable. Older adults in our research therefore appeared particularly critical of the validity of information on social media. One participant stated that he prefers to rely on specialists, “good sources of information,” since social media offers quick access to information that is not necessarily reliable:

“Who else is going to give me real information? Not Facebook, it’s not real, it’s not the so-called Facebook specialists with degrees as long as your arm. That’s where you come in, you do a Google search on them, and you can’t find them. That’s not where the real information is going to come from, so get informed, talk to your doctor, turn to WHO, turn to your government, we’ve got government specialists there… Caroline Quach, she’s no pushover. We have good sources of information, but the world wants to have fast information (P 1; 67 years old).

In short, many participants said they don’t pay attention to the kind of information associated with social media. For one participant, ignoring the misinformation circulating on social media reflected his general disinterest in these platforms: “Because there’s information out there for everyone, as the English term *fake news* says. So, since I’m not a big fan of social media, I wasn’t interested either. […]” (P 17; 68 years old). The same participant asserted that he doesn’t let himself be influenced by misinformation, and follows his own opinion, whatever the cost:

“And when they [social media users] said things about the pandemic, the vaccine or whatever, I let them talk, but that didn’t influence me […] I formed an opinion and then I said to myself, ‘I have an idea, it may not be the best one, but I tell myself I’m going to go all the way’, quite simply. (P 17;68 years old)

Then, among the participants using social media, many said they have unsubscribed or blocked people spreading misinformation, so as not to be exposed to it themselves. Referring to some of these people, one participant mentioned:

“[…] And their response was, “Well, you go ahead, and as soon as you’re all vaccinated, I won’t have to go. So I thought, “Okay, it’s the same as what you think, because your freedom is more important than your collective freedom,” and then it was “Okay, unsubscribe. I’m not stubborn with people who’ve gone that route, and there’s nothing that’s going to make them change their minds. […]. I used to have a certain number of friends on Facebook, and now I have a few less, because the ones who were… who were spreading false and misleading information, well, they’ve disappeared from my Facebook friends list.” (P 27; 62 years old).

One participant shared that blocking these people has helped her feel better in general:

“Then those who put on too much, well, I blocked them. […]. Because I don’t want anyone messing with my head, so I blocked them, you know, there are fanatics in everything […] So I blocked people on that, anti-covid, and my God, I’ve been feeling good ever since.” (P 33; 65 years old).

As it was highlighted previously, disregarding of ignoring statements from friends who share misinformation is also a strategy applied in face-to-face interactions.

Finally, to cope with information overload, some of the older adults interviewed mentioned setting up special information routines during the pandemic. Indeed, many said they stopped listening to the news all day long, but that they preserved a specific period in their daily schedule to get informed, whether it was the time of a radio program or the newscast. “We stopped listening to the news all day long; at a certain point, it’s too depressing, and we listened to it once at the end of the day, on TV,” shares one (P 14; 71 years old). This strategy consists in taking a step back and limiting the time devoted to news, so as not to be overwhelmed by it.

## Discussion

Very similar to the statistics on information sources consulted by older adults, the participants in our study favoured traditional media [43]. The fear, anxiety, stress and worry generated by misinformation and information overload are consistent with the findings of other studies [12, 14]. However, for the participants, these harmful effects of misinformation would affect those closest to them more, illustrating the third-person effect, i.e., the perception that others are more vulnerable to misinformation than the self, highlighted by other studies [44, 45]. Beyond negative emotions, our results reveal that misinformation contributes to polarization between individuals, sometimes leading to friendship breakdowns. Thus, it is social relationships that suffer from the conflicts caused by misinformation. For older adults, who may have a fragile social network [46], this issue can be crucial, as they risk finding themselves alone [47].

The susceptibility of older adults to online misinformation is based on the idea that they are “lagging behind” the rest of the population in their use of digital technology [23, 30, 48, 49]. However, the participants in our study claim to be able to spot misinformation easily, and although they are sometimes exposed to it, this does not mean that they adhere to it. In fact, our results show that older people are critical, active and make sure to implement strategies to protect themselves, regardless of their gender or level of education. These findings are in line with those of a study which found that older adults understand the necessity for a degree of caution while using the internet [50]. Based on these results, it seems appropriate to question the concept of vulnerability to misinformation and its association with older people. Indeed, while advanced age is negatively associated with internet skills [23, 29, 32], other factors also need to be taken into account, such as level of education, socio-economic position and user experience [23, 29]. One study reveals that, compared to the use of the internet for capital-enhancing activities, age is a less relevant variable than “autonomy of use” and the ability to surf the web [29]. In other words, the “age divide” in relation to digital media is often more complex than it seems as it involves multiple factors such as physical access, digital literacy, information and communication technologies utilization as well as socio-economic factors [29]. The results of our research seem to concur with the conclusion that age alone does not explain the ability to spot misinformation and suggest significant heterogeneity among older adults. It is crucial to be attuned to social and cultural variances among older people [51]. Differences within this group are all the more numerous as older adults have accumulated many life experiences [51], and the experience of aging varies from person to person [48]. In public health, standardizing approaches to older people can be harmful, as they tend to favour ageist approaches [33].

Lastly, several studies are alarmed by information avoidance [52, 53], which is a strategy for avoiding exposure to information deemed threatening or undesirable [54]. Establishing an information routine by targeting information sources to be consulted at specific times can, on the contrary, be seen as a healthy strategy for preserving mental health and better managing the information overload created in particular by the media, while remaining informed.

### Research and practice recommendations

Initiatives developed to combat information overload focus primarily on misinformation. However, our study suggests treating information overload as a distinct phenomenon, as this will enable us to rethink public health communication strategies and target the messages that are essential for limiting information overload.

Furthermore, our study reveals that health misinformation circulating on social media can not only generate negative emotions, but the polarization that breaks down dialogue and isolates individuals also appears to be crucial to health issues [55–57]. This is why it is essential to consider polarization in the development of public health interventions and communication strategies [58, 59].

Finally, the results of our study highlight the agency of older adults to implement strategies for managing misinformation and information overload. Our research illustrate the relevance of relying on older adults’ ability to act [20, 60, 61] for the development of public health interventions [62, 63].

This study has a number of limitations. Older adults living in residential facilities are often excluded from studies, due to the added difficulty of accessing this group. As these individuals are likely to have more physical and cognitive limitations, this aspect of recruitment taints research results, over-representing the realities of healthy older adults [48]. This is the case for this research, given that only one participant lived in a seniors’ residence. Indeed, this is a frequent limitation characterizing studies of internet use by older adults in general [48]. In addition, as recruitment was online this assumes that participants possessed some familiarity with the internet and computers. Another limitation of this study concerns the social desirability bias on the part of participants, characteristic of qualitative research. This bias was limited by the fact that the interviews were conducted by people trained in qualitative research. Qualitative researchers are trained to establish trust with participants. Moreover, they employ questioning techniques such as formulating open-ended questions and adopted an attitude of active listening during the interview.

## Conclusions

Our results show that, although older adults may have a facility for detecting misinformation, they are affected by polarization and information overload. We therefore suggest that communication strategies be developed to minimize polarization and information overload.

### Electronic supplementary material

Below is the link to the electronic supplementary material.

Supplementary Material 1

## Data Availability

The datasets generated and analysed during the current study are not publicly available due to the Centre intégré universitaire de santé et de services sociaux du Centre-Sud-de-l ’Île-de-Montréal (CIUSSS) Ethics Committee regulation. Data are available upon reasonable request. Inquiries for data access should be sent to the corresponding author who then contact the Centre intégré universitaire de santé et de services sociaux du Centre-Sud-de-l ’Île-de-Montréal (CIUSSS) Ethics Committee for permission to openly share the data.

## Abbreviations

WHO: World Health Organization (Organisation Mondiale de la santé, OMS)
CTRCAQ: Conference of the Regional Roundtables for Seniors in Quebec
CHSLD: Centres d’hébergement de soins de longue durée (residential and long-term care centres/residential centres)
CIUSSS: Centre-Sud-de-l’Île-de-Montréal
