# Supplementary material for: How older adults manage misinformation and information overload - A qualitative study
Source: BMC Public Health. 2024 Mar 21;24:871. doi: 10.1186/s12889-024-18335-x (PMC10956171; doi:10.1186/s12889-024-18335-x)
Supplement: Supplementary file 1 — Supplementary Material 1 [file 12889_2024_18335_MOESM1_ESM.pdf]

Research Study

COVID-19 and Infodemia: COVID-19 and infodemia: Informational Practices among communities in situation of vulnerability in Quebec during the COVID-19 Pandemic

Semi-Directed Interview Guide

[For the interviewer: Please go over all terms in the information and consent form with the participant and make sure everything is well understood.]

Thank you for agreeing to take part in this project. Our aim is to understand better how people have been getting information during the COVID-19 pandemic. This research will help improve how public health authorities communicate with people aged 60 and over, so they can better understand their information and health needs.

During this interview, I'll ask you about the sources of information you use, how you get that information, and how you decide if it's reliable or not.

The information you share during this interview will be kept private. I've signed a confidentiality agreement, as has every member of the research team. And when we share the results, your identity won't be revealed.

Feel free to speak openly. There are no right or wrong answers, and you don't have to answer every question. If you feel uncomfortable at any point, we can stop the interview.

Do you have any questions before we start?

Once the consent form has been signed and the participant has agreed to the interview being recorded, start the recording.

Or if it ***is an oral consent Start recording and ask inform consent question***

Opening question / general Information

1. Generally speaking, can you tell me how COVID-19 has impacted your health or your family's health? Can you give me some examples?

2. Did you have COVID-19? (If yes, explore how it happens, If yes, explore how it happened, how the participant was informed, and how they sought the information.)

Information sources

3. Since the beginning of the COVID-19 pandemic, how have you obtained information about the pandemic?

4. Since the beginning of the COVID-19 pandemic, how have you obtained information on preventive measures?

5. What are your favourite sources of information? Why?

6. What sources of information do you dislike? avoid? Why?

7. We've been receiving a lot of information about COVID-19. Can you name the organizations and people who send you information? What do you think about it?

8. How has the pandemic affected the way you seek information?

9. Which topics about the pandemic are you most interested in?

Information research strategies

10. What is the most recent information you sought about the COVID-19 pandemic? Where did you search for this information?

11. How do you search for information online about the pandemic and preventive measures? Can you give me an example?

12. Approximately how much time do you spend searching for information for information about the pandemic?

13. When you are on social media [Facebook, Twitter, Instagram, etc.], what kind of page, profile or groups do you consult to get information about the pandemic and preventive measures?

14. Generally speaking, what do you think about the modes of communication used by public health authorities [video, text, image, graphic, symbol, message radio, Emergency alert on our phones etc.]?

15. Would you rather search for written [papers, public panels, publications, etc.], oral [radio, podcast, etc.], or visual [TV show, documentaries, short videos, etc.] information?

16. Do you share the information you consult about the pandemic with other people?
    - If yes, what kind of information do you share? Why?
    - How do you share it?
    - How often?
    - With whom?

17. Approximately how much time do you spend consulting information for information about the pandemic?

Informational behaviours: Information reliability assessment and information understanding

18. Generally speaking, what do you think of the information you consult?

19. What information do you find difficult to understand? Do you have examples of information you didn't understand?

20. There is a lot of disinformation and fake news; what do you think about it?
    - *Do you have examples of false news?*
    - *What did you think about it?*
    - *How did you know it was false news?*

21. How do you know if information is reliable?

22. What do you think of the information provided by public health authorities?

23. How much do you trust the information provided by the government/your entourage [family, friends, social and medical personnel]?

24. Based on your experience, what is the most relevant media to obtain reliable information?

25. Generally speaking, what do you think of the information and awareness-raising campaigns of public health authorities?

26. Sometimes experts are not saying the same things, what do you think about this?

27. What do you think should be improved for you to be well informed?

Informational practices and health behaviours

How has the information and advice you have consulted influenced your health?

28. How has the information and advice you have consulted influenced your preventive practices [for example, requirement to wear a mask, physical distance, etc.]?

29. How has the information and advice you have consulted influenced your decisions related to vaccination?

30. What do you think of the amount of information produced about the pandemic? [explore the perceptions at the different stages of the pandemic]

At this point, revisit ambiguous or unclear answers and try to summarize the main elements of the interviews.

Conclusion

1. Is there anything we haven't talk about that you would like to mention.
2. Do you have any comments about this research project on the COVID-19 pandemic and researching information practices?
3. Verify information for sending monetary compensation.

Thank you!
